# Faces Divulge the Origins of Caribbean Prehistoric Inhabitants

**DOI:** 10.1038/s41598-019-56929-3

**Published:** 2020-01-10

**Authors:** Ann H. Ross, William F. Keegan, Michael P. Pateman, Colleen B. Young

**Affiliations:** 1 Department of Biological Sciences, North Carolina State University, Raleigh, NC 27695 USA; 2 Research Associate, National Museum of Natural History, Smithsonian Institution, Washington, DC 20013 USA; 3 Curator of Caribbean Archaeology, Florida Museum of Natural History, University of Florida, Gainesville, FL 32611 USA; 4 Director, Turks & Caicos National Museum, Grand Turk, Turks & Caicos Islands, Turks, USA; 5 Department of Anthropology, University of Missouri, Columbia, MO 65203 USA

**Keywords:** Biological anthropology, Archaeology

## Abstract

The origins of the first peoples to colonize the Caribbean Islands have been the subject of intense debate for over 30 years. Competing hypotheses have identified five separate migrations from the mainland with a separate debate concerning the colonization of The Bahamas. Significant differences in the facial morphology of the pre-Columbian inhabitants of Hispaniola and Cuba led to the present study of Lucayan skulls from The Bahamas. The goal was to determine which group the native Lucayans more closely resembled to resolve this long-standing dispute. The results indicate that they are related to groups from Hispaniola and Jamaica and not to Cuban inhabitants. This study clarified the larger picture of Caribbean migrations and supports evidence for a Carib invasion of the Greater Antilles around AD 800.

## Introduction

**Figure 1:**
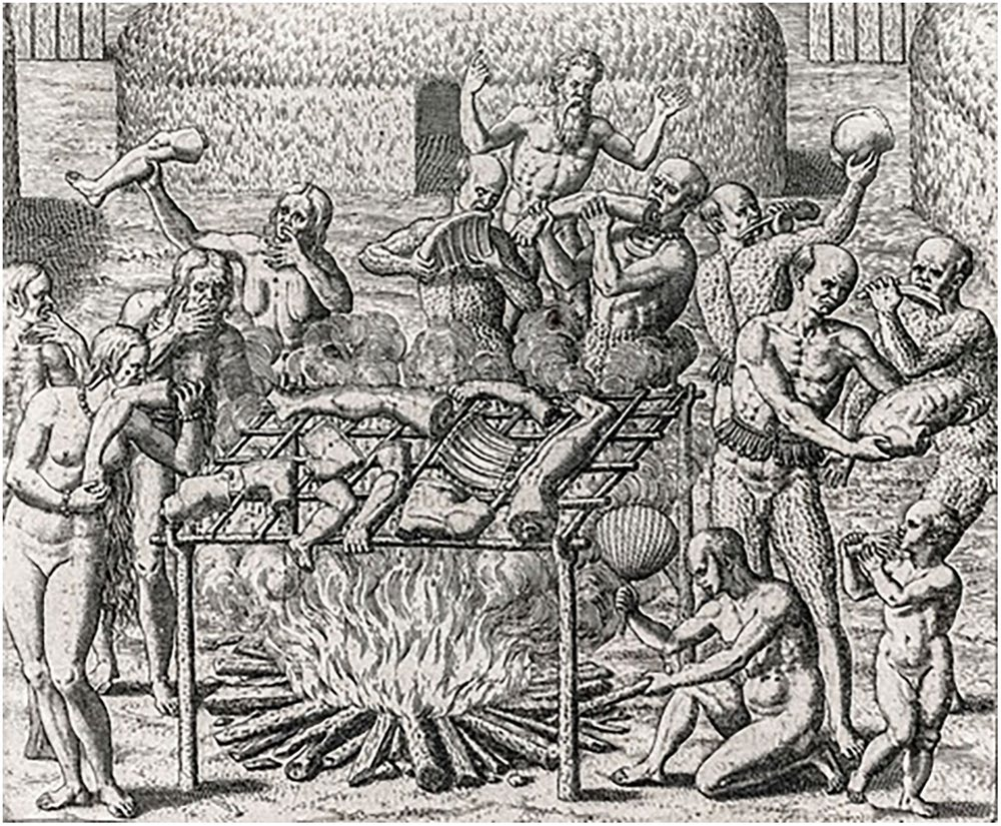
Cannibalism in Brazil by Theodor de Bry, 1596. [The author died in 1598, so this work is in the public domain in its country of origin and other countries and areas where the copyright term is the author’s life plus 100 years or less].

The defining image of the Columbian encounter is ravenous cannibals descending upon unsuspecting peaceful Arawak villages, whence they ate the men and took the women as wives (Fig. 1). From the moment he landed on the first Bahamian island – Guanahaní – Columbus wrote, “I saw some who had marks of wounds on their bodies and I made signs to them asking what they were, and they showed me how people from other islands nearby came there and tried to take them, and how they defended themselves; and I believed and believe that they come from Tierra Firme to take them captive”^1^. This is the first of ten allusions to Carib raids during the first voyage^2^. Archaeologists have questioned this assertion based on the possible confusion of Caribe and Caniba (the Asiatic subjects of the Grand Khan), content with the knowledge that the true Caribs never advanced further than Guadeloupe in the Lesser Antilles^3,4^.

The Caribbean archipelago extends almost 3,000 km from the mouth of the Orinoco River in northern South America to Florida and the Yucatán enclosing the Caribbean Sea with three major island groupings consisting of the Lesser Antilles, the Greater Antilles, and The Bahamas^5^. The Caribbean’s stepping stone arrangement facilitated human and animal dispersals into the islands throughout prehistory.

The first and still popular framework for the peopling of the Caribbean by small groups of hunters, fishers, and foragers most likely infiltrated the archipelago by canoe was based on technological differences^4,6,7^ that identified a “Lithic Age” (flaked-stone technology) migration as crossing the Yucatán passage from Central America to Hispaniola and Cuba between 4000–5000 BC, an “Archaic Age” (ground-stone technology) migration from Trinidad to the Greater Antilles through the Lesser Antilles around 2500 BC, and a “Ceramic Age” (Saladoid series pottery) migration from the mouth of the Orinoco River (Venezuela/Guiana) through the Lesser Antilles to Puerto Rico around 500 BC. After reaching Puerto Rico there was a 1,000-year hiatus before Ceramic Age expansion resumed with the colonization of Hispaniola, Jamaica, Cuba, and The Bahamas.

To date, the different proposed migration routes have been largely based on archaeological evidence of similarity and dissimilarity of pottery styles, which has provoked heated debate within the discipline^3,4,7–13^. The Bahamas was the last of the Caribbean archipelagoes to be settled by humans. The earliest evidence of human arrival is the well-dated Coralie site on Grand Turk, Turks & Caicos Islands indicate initial occupation in the early 8th century AD^14^. Pottery analysis showed different materials mixed in the clay that was not endemic to the Bahamas suggest it was brought to the Turks & Caicos from Hispaniola^15^. This evidence supported the conclusion that the archipelago was first colonized by people from Hispaniola^16,17^. The name originally used by the Spanish to describe the Bahamas was used to argue that the Lucayans originally came from Cuba^18^. Early radiocarbon dates obtained from three Lucayan sites in the central Bahamas (i.e., New Providence, northern Eleuthera, and San Salvador) supported this Cuban connection^19,20^.

Ancient DNA data suggests a northern South American origin for initial dispersal into the Caribbean^21^ yet it is based on a single individual and should be viewed with caution. Craniofacial morphology (e.g., craniometrics) has been empirically demonstrated as a suitable genetic proxy for examining population structure as cranial data have a greater spatial and temporal coverage than ancient DNA from pre-Contact New World populations^22,23^. The cranial base, facial and neurocranial modules are integrated systems that maintain stability throughout the whole complex during growth and development with the neurocranium being more susceptible to phenotypic plasticity^23,24^.

Studies of population diffusion into the Caribbean have been neglected until recently, partly due to the scarcity of available skeletal material resulting from unfavorable preservation environments and thus, there are significant gaps in knowledge^22,25–29^. Here, we propose evidence for multiple dispersal events and ancestral origins of Pre-Contact Caribbean Amerindians employing facial morphology as a genetic proxy.

## Results

**Figure 2:**
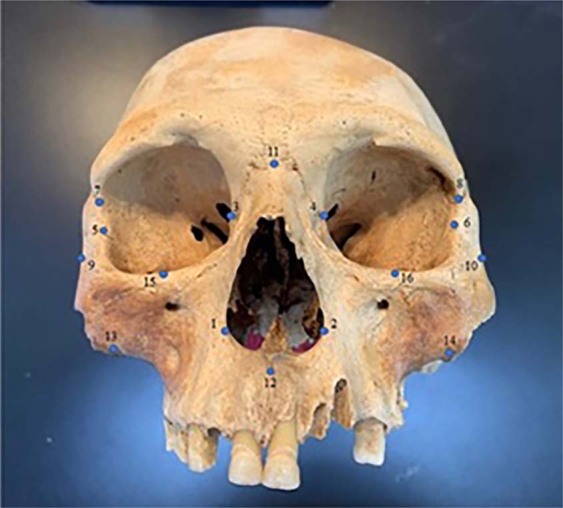
Sixteen homologous anatomical landmarks used in this study.

Three migration routes are evident in a sample of 103 individuals from 10 localities using 16 homologous anatomical landmarks (Fig. 2, Tables 1 and 2, see Materials and Methods section; raw coordinate data are available in the Supplementary File).

**Table 1:** Homologous anatomical landmarks included in this study.

1, 2 Alare right/left	11 Nasion
3, 4 Dacryon right/left	12 Subspinale
5, 6 Ectochonchion right/left	13, 14 Zygomaxillare right/left
7, 8 Frontomalare temporale right/left	15, 16 Zygoorbitale right/left
9, 10 Jugale right/left

**Table 2:** Sample composition and provenience.

Sample	N	Provenience
Bahamas	8	(ca. 1000–1500 A.D.) National Museum of the Bahamas’ Antiquities
Colombia	5	Pre-Contact, American Museum of Natural History
Cuba	21	(ca. 800–1500 A.D.) Museo de Montane, Havana, Cuba and National Museum of Natural History
Hispaniola	15	(ca. 800–1500 A.D.) National Museum of Natural History
Jamaica	7	(ca. 800–1500 A.D.) National Museum of Natural History
Yucatan, Mexico	12	Chichen-Itza, Peabody Museum, Harvard
Panama	6	Pre-Contact, Patronato Panama Viejo, Panama
Puerto Rico	10	(ca. 800–1542 A.D.) American Museum of Natural History
Florida	15	(ca. 1300–1400 A.D.) American Museum of Natural History
Venezuela	4	Pre-Contact, American Museum of Natural History

### Geometric morphometrics

**Figure 3:**
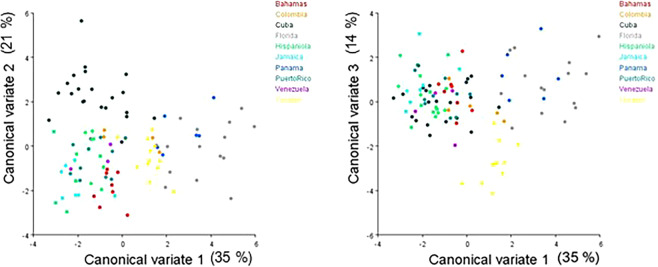
Scatter plots of the first three canonical variates (accounting for 70% of the total shape variation).

The Procrustes ANOVA results show significant group variation for shape (F (369, 3813) = 2.27, p =  < 0.0001) and centroid size (F(9,93) = 737.92, p =  < 0.0001). The canonical variates analysis (CVA) produced nine significant canonical axes and the eigenvalues indicate that 35% of the shape variation is accounted for on the first canonical axis, 21% on the second canonical axis, and 14% on the third axis for 70% of the total shape variation (Fig. 3). The Mahalanobis distance (*D*) matrix, a measure of biological distance, shows that the two closest groups are Hispaniola and Jamaica, followed by Puerto Rico and Venezuela, Hispaniola and the Bahamas, with the most dissimilar groups being Panama and The Bahamas, Hispaniola, and Jamaica. (Table 3).

**Table 3:** Mahalanobis distances showing the shape change between all pairs of groups.

	Bahamas	Colombia	Cuba	Florida	Hispaniola	Jamaica	Panama	PuertoRico	Venezuela	Yucatan
Bahamas	0									
Colombia	8.7213	0								
Cuba	7.0197	7.2287	0							
FL	7.4521	8.0386	6.584	0						
Hispaniola	4.9188	6.5737	6.5664	8.2246	0					
Jamaica	6.2524	7.8868	8.19	8.9742	3.9442	0				
Panama	9.6924	8.0138	8.3187	6.1025	9.6082	9.5254	0			
PuertoRico	6.9211	6.5522	6.9993	7.5231	5.7005	5.0428	7.4326	0		
Venezuela	8.0587	6.2165	8.0882	8.7086	6.4041	6.2769	8.5496	4.8494	0	
Yucatan	7.1968	7.2409	6.8812	6.6578	7.0205	8.2154	8.0951	6.324	7.106	0

All groups were significantly different with *p*-values ranging from 0.01 to < 0.0001 level based on 10,000 permutations.

**Figure 4:**
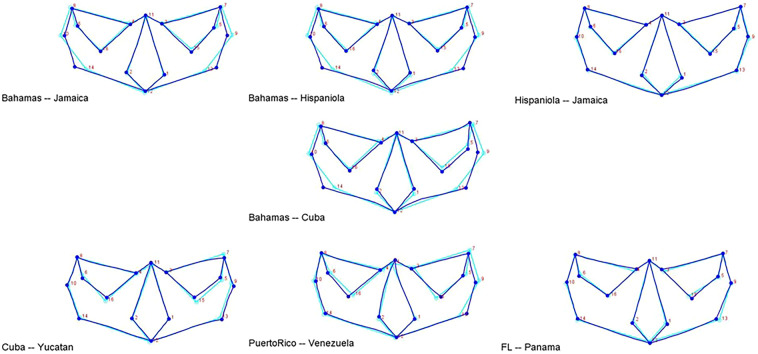
Wireframes showing the direction and shape change between one consensus configuration and the other showing facial breadth variation among The Bahamas, Hispaniola, and Jamaica samples, the variation between Cuba and the Yucatán, which is associated with orbital shape. The variation between Puerto Rico and Venezuela is related to orbital shape. And the global morphological similarity is evident between Hispaniola and Jamaica and between the samples from Florida and Panama.

Morphological variation is illustrated via wireframe graphs that depict the magnitude and direction of shape change between two mean configurations generated using the discriminant function analysis (Fig. 4). The groups illustrated were selected according to the clusters produced by the hierarchical cluster analysis outlined in the next section. The variation between The Bahamas and Hispaniola and Jamaica samples primarily involves those landmarks related to facial breadth. The variation between The Bahamas and Cuba includes overall facial morphology, while the variation between Cuba and the Yucatán is associated with orbital shape. The variation between Puerto Rico and Venezuela is also linked to orbital shape (e.g., zygoorbitale is more inferiorly and laterally placed in the Puerto Rican sample). The global morphological similarity is evident between Hispaniola and Jamaica and likewise, between the samples from Florida and Panama.

### Hierarchical cluster analysis

**Figure 5:**
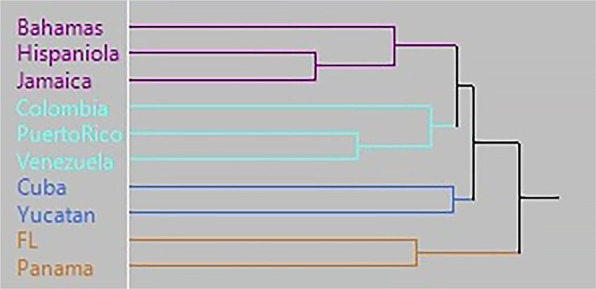
Dendrogram produced from the hierarchical cluster analysis showing 4 clusters. One cluster (purple) groups Hispaniola, Jamaica, and the Bahamas samples. The second cluster (teal), groups Puerto Rico, Venezuela, and Colombian samples. The third cluster groups Cuba and Yucatan and the fourth cluster groups Florida and Panama.

**Figure 6:**
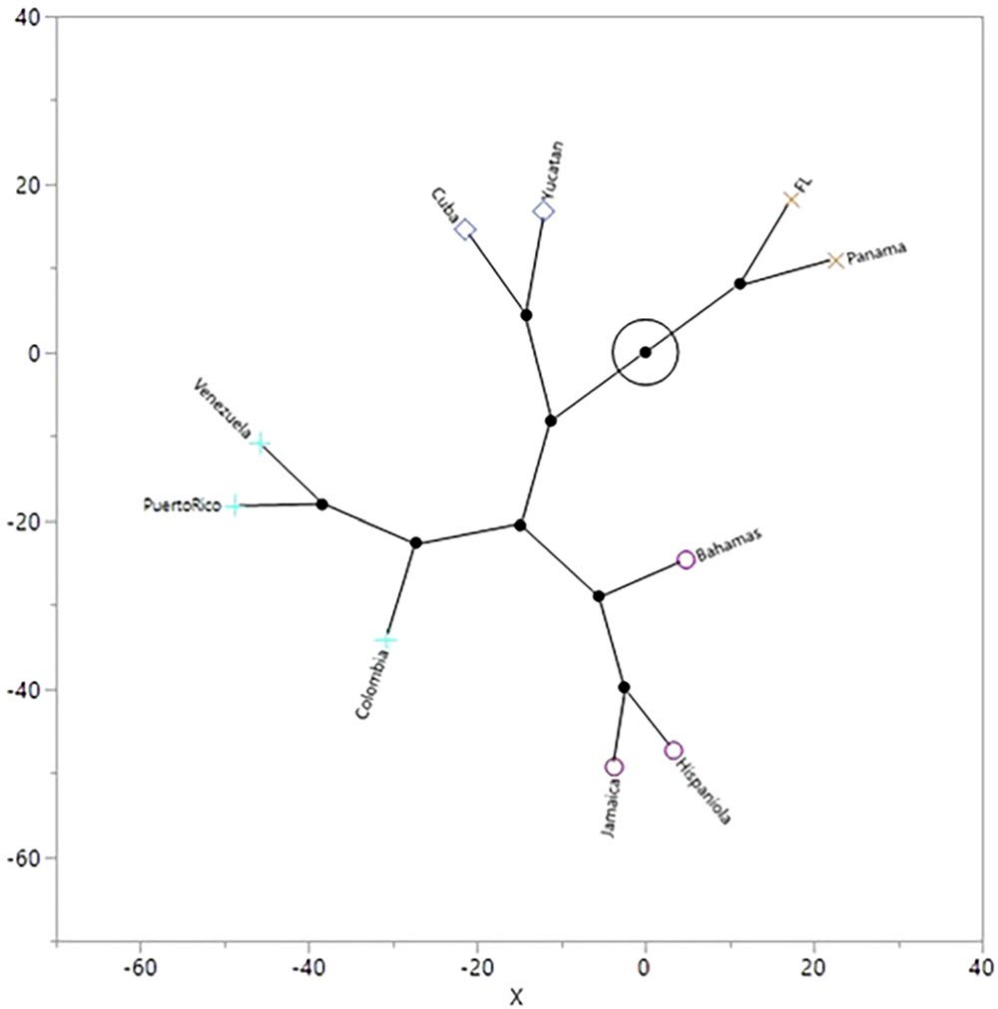
The constellation plot, which arranges the samples as endpoints, was also produced by the hierarchical cluster analysis. The length of a line between cluster joins represents the distance between the cluster joins. The plot illustrates that the most distinct groups are Florida and Panama and shows that this cluster is the most distant from the clusters on the lower half of the plot (purple and teal) and the cluster on the upper half (blue) of the plot is about the same distance from the Panama/Florida cluster as it is from the clusters on the lower half of the plot.

The dendrogram produced by the agglomerative cluster analysis using the Mahalanobis distance matrix shows four distinct clusters (Fig. 5): (1) Panama/Florida, (2) Yucatán/Cuba (initial colonization), (3) Colombia/Venezuela/Puerto Rico (Arawak expansion), and (4) Hispaniola/Jamaica/Bahamas (Carib invasion). The constellation plot clarifies the biological relationship among the clusters and shows that Cuba clusters with the Yucatán sample, which is twice as distant as the other two Caribbean clusters (purple and teal) are to each other (Fig. 6).

### Spatial analysis

**Figure 7:**
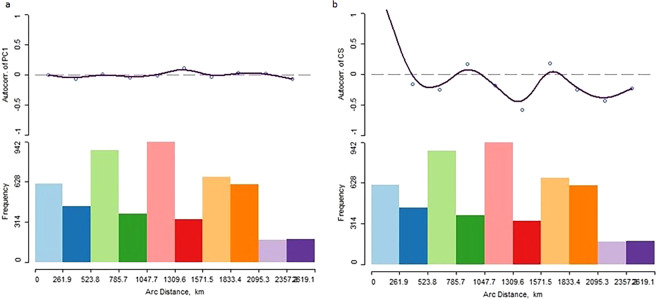
Correlograms for PC1 (**a**) and centroid size (**b**) depicting the lack of spatial autocorrelation for shape and a non-monotonic decrease in centroid size with increasing distance.

The spatial autocorrelation for shape based on the first principal component and centroid size does not show a spatial association for morphological shape but does show a strong positive spatial association for centroid size (Table 4). The correlogram (Moran’s *I* by kilometers) for shape (PC1) shows no spatial structure while the correlogram for centroid size shows the autocorrelations decreasing with increasing distance (Fig. 7a,b). However, the decrease is not monotonic.

**Table 4:** Spatial autocorrelation shows no spatial association for shape but does for centroid size.

Variable	Coefficient	Observed	Expected	Std Dev	Z	Pr > Z
PC1	Moran’s *I*	−0.0022	−0.0098	0.0288	−0.264	0.7920
	Geary’s *c*	0.6921	1.00000	0.2968	−1.037	0.2995
CS	Moran’s *I*	1.1458	−0.0098	0.0366	31.5	<0.0001
	Geary’s *c*	0.0028	1.00000	0.0975	−10.2	<0.0001

## Discussion

Because on average, phenotypic traits and global patterns of craniometric variation are consistent with neutral evolutionary forces such as genetic drift and flow, inferences can be made regarding past population history and structure including origins and demic diffusion using craniofacial morphology^30^. In a recent regional approach of pre-Contact New World craniofacial variation, a strong positive spatial association on morphology was found for both shape and size suggesting that these New World Amerindians were heterogeneous, which is consistent with an isolation-by-distant model after initial diffusion^22^. The results of the present study, per contra, show that Caribbean Amerindians were not spatially patterned (i.e., for shape, Fig. 6a) suggesting homogeneity rather than heterogeneity of the populations. Population homogeneity can arise by the migration of cohesive demes (or breeding populations) who are genetically different that settles in an area within the local population^31^. And while a significant spatial pattern (Table 4) was observed for centroid size, it did not show a monotonic decrease with distance or a clinal pattern (Fig. 6b) as would be expected under an isolation-by-distance model similar to kinship^32^. The morphological variation and similarity and spatial patterning observed in this study reasonably reflect repeated population migrations and expansions by various groups from different directions.

Contacts between Florida and the islands have long been proposed^33–35^ as have connections between the Isthmo-Colombian region^36^. However, the individuals from Florida and Panama in our sample show no clear relationship with individuals from the islands. Archaeological evidence documents a strong relationship between Venezuela and Puerto Rico beginning, at least, with the movement of Saladoid pottery into the islands between 800 and 200 BC. Our data confirm a biological relationship between individuals from Venezuela and Puerto Rico. However, the movement of peoples between the islands and mainland, and between the islands aligned from Trinidad to Puerto Rico, is far more complicated than origins alone can resolve^3^. Similarities in stone-tool technology observed in Belize and Cuba have been used to identify the Yucatán Passage as the first crossing of humans from the mainland to the islands^37,38^. Our data support this association, especially given the significant differences between individuals from Cuba and those from other islands in the Greater Antilles. Differences in facial morphology between Cuba and The Bahamas further support that the Lucayans did not originate in Cuba. The earliest Ceramic Age settlements on Hispaniola and Jamaica are dated to around AD 600^3^. They are few in number, widely scattered, and are recognized by the presence of “redware” pottery, associated with Archaic Age communities^2^. Meillacoid style pottery appears suddenly in Hispaniola around AD 800 and was brought to Jamaica (circa AD 900) and The Bahamas (circa AD 1000) during population expansion into essentially unoccupied territories. Meillacoid pottery also reaches eastern Cuba around AD 1000^39^. Diffusion into Cuba is different because it reflects the infiltration of long-established Archaic Age communities, which would explain why individuals in Cuba are morphologically different from their neighbors. Meillacoid pottery is identical to the pottery associated with the Carib expansion^13^. Commencing in the northwestern Amazon basin around AD 500, the timing is appropriate for the sudden appearance around AD 800 of this new pottery style in Hispaniola^10,11^. Their arrival clarifies why population expansion suddenly resumed after a 1,000-year hiatus. The Carib invaders were on the move, as attested to their rapid expansion across South America and also resolves Columbus’ often perplexing descriptions of fierce raiders in The Bahamas, Jamaica, and western Hispaniola. Raiders that Las Casas would identify in a marginal note to Columbus’ Diario as “Caribes”^1^.

Genetic evidence eventually may help to verify or refute our proposed Carib migration hypothesis. However, there are currently three main problems. First, too few studies have been completed, although we would note that our Bahamas samples are part of an ongoing study of genetic relationships in the circum-Caribbean. Second, community-level identifiers are not yet available, it is difficult to evaluate the impacts of admixture over time^21,40^, and a high degree of genetic variability has been reported for the northwest Amazon homeland of the Carib migrants^41,42^. Third, previous studies have looked only at the two generally accepted Archaic Age and Arawak/Taíno expansions, with some reference to the centuries later Carib migration into the Lesser Antilles^27^. If future studies do not look for Carib influences, they will produce incomplete results.

Nevertheless, several studies support our conclusions. The DNA analysis of Saladoid individuals strongly suggest a common origin and genetic continuity over time^27^, although genetic contributions in Puerto Rican individuals that are more closely related to northwestern South America has been recognized^40^; and the study of dental morphology identified a distinct Cuban cluster^25^. These results independently confirm the Puerto Rico-Venezuela and Cuba-Yucatán clusters identified here. Finally, “marked haplogroup variation seems to be present among the three neighboring Caribbean islands (Puerto Rico, Hispaniola, and Cuba),” such that colonization of the Caribbean was mainly due to successive migration movements from mainland South America in different time periods^26^. Haplogroup diversity among the three islands and successive waves of migrations are exactly what our data predict. Finally, guinea pigs (*Cavia porcellus*) first appeared in the Antilles “sometime after AD 500,” and their origins were genetically traced to northwestern South America^43^. Their translocation occurred at the same time and from the same place that we propose for the Carib expansion into the Caribbean islands.

**Figure 8:**
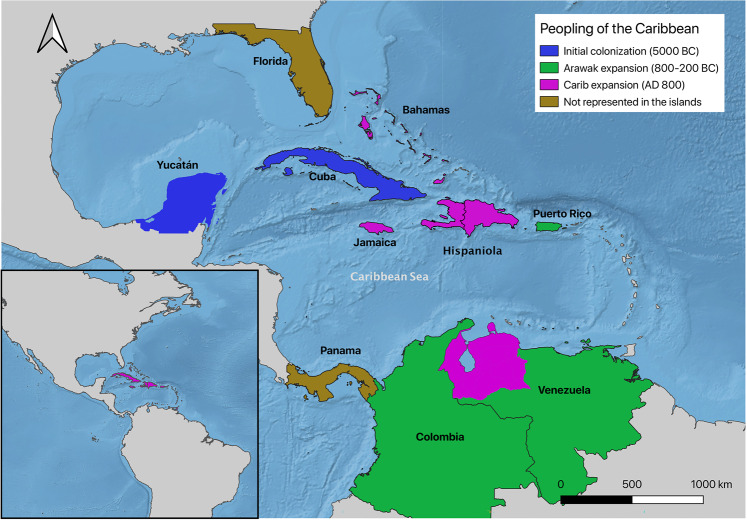
Proposed new three migration routes for the peopling of the Caribbean. [The map was generated using public domain free raster + vector data from @naturalearth.com].

The reasons that most archaeologists failed to make this connection is that Rouse^4^ was adamant that there was only one Ceramic Age population expansion, archaeologists were fixated on the shortest geographical water crossings, especially the “stepping-stone” Lesser Antilles^44^, and interest in Island Caribs was focused on the 17th century testimony of French missionaries living in the eastern Caribbean. It is only in the past decade that archaeologists have recognized the pre-Columbian capacity to directly cross the Caribbean Sea^3,28,45^. Looking at faces, instead of islands, we see three distinct populations from three distinct places (Fig. 5). The first was the initial peopling of Cuba and the northern Antilles across the Yucatán Passage around 5000 BC. Next was the arrival of Arawak-speaking peoples in Puerto Rico from coastal Venezuela between 800 and 200 BC. Finally, Carib colonists crossed the Caribbean Sea to arrive in Hispaniola around AD 800 and then continue their rapid expansion into Jamaica and The Bahamas (Fig. 8). The evidence highlights a completely new perspective on the people and peopling of the Caribbean.

## Materials and Methods

### Samples

Samples were selected for their availability at various museums and excellent preservation. The samples used in this study total 103 individuals from the Caribbean and pre-Contact Amerindian samples from Florida and Panama for comparison. The Caribbean samples from Jamaica, Hispaniola, and Puerto Rico have all been temporally identified as Ceramic Age or Taíno of the Arawak language group. The sample from Cuba was also archaeologically identified as Ceramic Age. The samples from The Bahamas have radiocarbon dates ranging from AD1000-1500. Regrettably, there is little contextual information available for the other museum samples from Venezuela, Colombia, and Florida. The sample composition and provenience are presented in Table 1.

For this project, we selected 16 homologous facial landmarks that should reflect the among group variation (Table 2 and Fig. 1). Only facial landmarks were utilized because these populations practiced intentional cranial modification and facial landmarks have been found not to be affected by cranial vault modification^24,46^. A Microscribe G2X and *i* digitizer were utilized to obtain the x,y, and z coordinates for each anatomical landmark. Due to the nature of prehistoric skeletal preservation from tropical areas, some of the sample sizes are small.

### Geometric morphometrics

Because skeletal preservation is a problem in archaeological samples, data had to be imputed in some of the individuals in order to increase sample sizes and retain maximum morphological coverage but no more than 20% (or 3 landmarks) were imputed for any given individual. Data were imputed using the software Morpheus *et al*. Java Edition using the GPA mean substitution function^47^. The mean substitution method was selected over other methods for estimating missing data as it has been found to produce a better fit to the original eigenvectors in human samples^48^.

Coordinate data must first undergo a Generalized Procrustes analysis or GPA transformation before subsequent statistical analyses can be performed. GPA translates, rotates, and scales each specimen and brings all individuals into a common coordinate system. Shape is defined as all of the geometric information that remains after the effects of location, scale, and rotational effects are removed^49,50^. Centroid size is a measure of geometric scale that is mathematically independent of shape^50^. The GPA procedure was performed using MorphoJ, which is freely available for downloading and developed by Klingenberg^51^. A principal component analysis (PCA) of the covariance matrix was conducted on the GPA transformed coordinates to reduce the dimensionality of the data for subsequent multivariate statistical analyses^50^. To examine shape and size (e.g. Centroid Size) variation among the groups, a Procrustes ANOVA was performed using principal component scores calculated from the PCA^51^. A canonical variates analysis (CVA) (with 10,000 permutation rounds that resulted in 450,000 pairwise comparisons) was performed to account for the maximum amount of among-group variance relative to within-group variance, which is also uncorrelated within and among groups. MorphoJ uses the standard equation for calculating the number of pairwise comparisons, which is k(k − 1)/2 with k = number of groups. CVA is used to examine variation for more than two groups known a priori that presents the most variation with the least dimensions possible^51^. Mahalanobis distance or generalized distance, which considers the correlations among variables when computing the distance between means, was used to examine group relatedness (Table 3)^51^. To visualize morphological variation between the groups the Discriminant Function Analysis (DFA) was used that calculates separate pairwise comparisons (with 1000 permutation runs) between groups using crossvalidation or n-1 method.

### Hierarchical cluster analysis

An average linkage hierarchical (or agglomerative) cluster analysis was conducted using the generalized distance matrix to examine group similarity and are commonly used in population history and structure studies^30,31^. Hierarchical clustering begins with every sample in a single cluster, then in each successive iteration, it merges the closest pair of clusters (distances between all pairs and averages all these distances) until all the data is in one cluster. The cluster analysis was performed in JMP ® Pro 14^52^.

### Spatial analysis

Moran’s *I*, a product-moment coefficient, and Geary’s *c*, a distance-type coefficient, were used to measure the spatial autocorrelation of shape (PC1) and centroid size. The autocorrelation is a measure of genetic similarity between individuals with reference to geographic separation (latitude/longitude). This procedure was performed using the Proc Variogram procedure in SAS 9.4, which reports Moran’s *I* and Geary’s *c* as a standardized z-score with a positive autocorrelation indicated by a ZI > 0 and Zc < 0^53^. Spatial correlograms were computed for shape (PC1) and centroid size to evaluate the spatial autocorrelation coefficients for all pairs of localities at specified geographic distance classes^54^. The correlograms were performed using the freeware software GeoDa v1.14.0^55^.

## Supplementary information

Supplementary Information

## Data Availability

Raw coordinate data (with accession codes) are available in the online version of this work.
